# Editorial: Applications of long-read sequencing in plant genomics and transcriptomics

**DOI:** 10.3389/fpls.2023.1141429

**Published:** 2023-01-31

**Authors:** Agata Daszkowska-Golec, Martin Mascher, Runxuan Zhang

**Affiliations:** ^1^ Institute of Biology, Biotechnology and Environmental Protection, University of Silesia in Katowice, Katowice, Poland; ^2^ Leibniz Institute of Plant Genetics and Crop Plant Research (IPK), Gatersleben, Seeland, Germany; ^3^ German Centre for Integrative Biodiversity Research (iDiv) Halle-Jena-Leipzig, Leipzig, Germany; ^4^ The James Hutton Institute, Dundee, United Kingdom

**Keywords:** long-read sequencing, plant genomics, transcriptomics, RNA-Seq, bioinformatics

Long-read sequencing technologies, such as PacBio and Oxford Nanopore sequencing, have enabled the development of new strategies and tools to study plant genomics and transcriptomics. These technologies have been used to improve genome assemblies in repetitive regions, to identify DNA and RNA modifications, and to detect alternative splicing (AS), alternative polyadenylation, and alternative transcription initiation sites, among other applications.

Zhang et al. demonstrated that even though in 2011, the draft reference genome of *Brassica rapa* ssp. *pekinensis* (Chinese cabbage) was completed and updated twice, the comprehensive genome annotation was still missing. Zhang et al. used PacBio technology to improve gene models and produced the annotated genome version 3.5. Moreover, this approach uncovered 830 novel protein-coding genes, defined UTRs for 20,340 annotated genes, identified 28,564 AS events, and 1,480 long non-coding RNAs. A more complete and high-quality reference transcriptome for *B. rapa* was created. It opens up new possibilities for further functional genomic research.

*Cunninghamia lanceolata*, commonly known as Chinese fir, is an essential timber species that contributes to 20-30% of raw materials to that country’s timber industry. Hu et al. conducted PacBio Iso-seq and RNA-seq analyses to investigate full-length mRNA transcripts and regulatory mechanisms behind cellulose and lignin biosynthesis. It led to the identification of 48,846 high-quality, full-length transcripts. Additionally, Hu et al. identified 18,714 differentially expressed genes (DEGs), of which28 and 125 DEGs were involved in cellulose and lignin biosynthesis. Moreover, 57 transcription factors (TFs) were identified. Additionally, their findings suggest that a similar regulatory network of secondary cell wall formation may exist in gymnosperms. Further, qRT-PCR was also used to investigate eight specific TFs related to compression wood formation. Overall, these findings provide valuable insights for molecular genetics research.

Senescence is a necessary part of plant growth and development. Early senescence in rice can result in a reduced yield and quality. The mechanisms behind this process need to be clarified. Sun et al. studied a rice mutant called *zj-es* derived from the japonica rice cultivar Zhejing22 and displayed early senescence symptoms, such as collapsed chloroplast, lesions in leaves, decreased fertility, dwarfism, and lower agronomic traits. The *ZJ-ES* gene was mapped in a 458 kb-interval between the markers RM5992 and RM5813 on Chromosome 3, suggesting it is a novel gene controlling early senescence. Whole-transcriptome RNA sequencing was done on *zj-es* and its wild-type rice to explore the mechanisms of early senescence. 10,085 differentially expressed mRNAs, 1,253 differentially expressed lncRNAs, and 614 differentially expressed miRNAs were identified. One of the modules (defined by Sun et. al. as the ‘turquoise’ one)in the weighted gene co-expression network analysis (WGCNA) was found to be the key to the occurrence of early senescence. The network of competing endogenous RNA (CeRNA) revealed 14 lncRNAs, possibly regulated by 16 co-expressed mRNAs through 8 miRNAs. Interestingly, enrichment analysis of DE genes and their DEmiRNAs targets revealed the importance of ROS-triggered autophagy-related pathways in early senescence in *zj-es* mutant.

In the last article collected on this Research Topic Liang et al. The study assembled the full-length chloroplast genome of Dongxiang wild rice line 159 (DXWR159), a Chinese common wild rice. The main findings were the Copy Number Variation (CNV) of Simple Sequence Repeats (STRs) between individuals and two significant Large Structural Variations (SVs) at the elementary-region level. The study also proposed a homologous recombination model to explain the formation of the two large SVs and confirmed that SSC switching frequently occurs in plant cpDNAs and that further investigation of the molecular mechanism underlying SSC switching may reveal new driving forces for large SVs. The study also recommends that researchers assemble the reference cpDNA using the species’ SSC-F structure rather than using their samples’ dominant structure.

This collection of articles shows that long-read sequencing has helped to advance plant biology and agricultural research in many areas. It includes studies that have applied the long-read technology as well as technical reports focusing on the challenges in analyzing long-read data, such as high error rates and limited coverage. Additionally, it looks at the unique challenges in plant species, such as the need for genomic resources and high-quality annotations for many non-model plants.

## Author contributions

AD-G wrote the first version of manuscript. All authors revised the manuscript and gave final approval for publication.

